# DNA oxidative damage in oral cancer: 8-hydroxy-2´-deoxyguanosine immunoexpression assessment

**DOI:** 10.4317/medoral.25924

**Published:** 2023-07-10

**Authors:** Jose Roberto Prieto-Correa, Ronell Bologna-Molina, Rogelio González-González, Nelly Molina-Frechero, Juan José Soto-Ávila, Mario Isiordia-Espinoza, Mariana Cristina Barrón Márquez, Sandra López Verdín

**Affiliations:** 1 Science Doctorate in Molecular Biology and Medicine. Molecular Biology Department, University Center of Health Sciences, Universidad de Guadalajara, México; 2 Molecular Pathology Area, Faculty of Dentistry, Universidad de la República, Montevideo, Uruguay; Department of Research, School of Dentistry, Universidad Juárez del Estado de Durango, Durango, Mexico; 3 Department of Research, School of Dentistry, Universidad Juárez del Estado de Durango, Durango, Mexico; 4 Department of Health Care, Universidad Autónoma Metropolitana, Xochimilco, Mexico City, Mexico; 5 Head and Neck Oncological Surgery Service, Instituto Jalisciense de Cancerología, Jalisco, México; 6 Institute of Research in Medical Sciences, Department of Clinics, Los Altos University Center, Universidad de Guadalajara, Jalisco, Mexico; 7 Master in Oral Pathology and Medicine. Department of Integral Dental Clinics, University Center of Health Sciences, University of Guadalajara, Guadalajara, Mexico; 8 Institute of Research in Dentistry, Department of Integral Dental Clinics, University Center of Health Sciences, University of Guadalajara, Guadalajara, Mexico

## Abstract

**Background:**

The development and establishment of oral squamous cell carcinoma are confined to carcinogenesis, which involves oxidative stress via oxygen-free radical production as a hydroxyl radical (HO•), considered the most important cause of oxidative damage to basic biomolecules since it targets DNA strands. 8-Hydroxy-2´-deoxyguanosine (8-OHdG) is considered a free radical with a promutagenic capacity due to its ability to pair with adenosine instead of cytosine during replication.

**Material and Methods:**

We collected 30 paraffin-embedded tissue samples of OSCC from patients treated between 2013 and 2018. We recorded risk habits, disease stage, disease free survival and death with at least 3 years of follow-up. 8-Hydroxyguanosine was evaluated by immunohistochemistry and subsequently classified as weak-moderate or strong positive expression. Additionally, we noted whether it was expressed in the cytoplasm and/or nucleus.

**Results:**

Most of the cases expressed 8-OHdG with a strong intensity (80%). All neoplastic cells were preferentially stained in only the cytoplasm (70.0%), but nuclear positivity was found in 30%, independent of the intensity. Based on the location in the cytoplasm and/or nucleus, tumors >4 cm showed a high frequency (95.5%) of 8-OHdG expression in only the cytoplasm, with a significant difference (*p value* 0.001). Additionally, overall survival was affected when immunoexpression was present in the cytoplasm and nucleus because all deaths were in this group were statistically significant (*p value* = 0.001).

**Conclusions:**

All tumors showed DNA oxidative damage, and 8-OHdG was preferentially expressed in the cytoplasm. This finding was associated with tumor size and, when present in the nucleus, might also be related to death.

** Key words:**DNA oxidative damage, oral squamous cell carcinoma, free radical.

## Introduction

According to the World Health Organization (WHO), cancer causes more deaths than any other disease (1). In 2020, GLOBOCAN (2) reported that lip and oral cavity cancer, with respect to other types of cancer, has the highest incidence and mortality worldwide, mainly in emerging countries. The International Agency for Research on Cancer (IARC) reports that squamous cell cancer registries reflect the effects of risk, genetic and environmental factors to which the population is exposed in developed or underdeveloped areas (3).

Oral squamous cell carcinoma (OSCC) is the most common histological type of cancer in the oral cavity (4), and its development is related to progressive damage to different cellular structures, as well as an imbalance in the ability to repair damage, a long process known as carcinogenesis (5). Tissue damage can be promoted by oxidative stress, which is defined as an increasing level of free radicals; as a consequence, an imbalance in intracellular homeostasis is present. If this damage progresses and is not stopped, some free radical molecules can reach DNA nitrogen bases (6).

Studies have clearly shown the participation of excessive endogenous and exogenous reactive oxygen species (ROS) production (e.g., related to risk habits or oxygen metabolism) via oxygen-free radical production as hydroxyl radicals (HO•), which are considered the most important causes of damage to basic biomolecules (proteins, membrane lipids, and DNA) (7-11). HO• attack DNA strands when produced adjacent to cellular and mitochondrial DNA, causing the addition of DNA bases to new radicals and leading to the generation of a variety of oxidation products (6).

DNA strand interaction with HO• preferentially occurs at the nitrogen base guanine because it has a lower oxidation potential, leading to the formation of C8-hydroguanine (8-OHGua) or its nucleoside form deoxyguanosine (8-hydroxy-2´-deoxyguanosine). For this product, the reaction of HO• addition leads to the generation of radical adducts; then, through one electron abstraction, 8-hydroxy-2´-deoxyguanosine (8-OHdG) is formed. Its promutagenic capacity is due to its ability to pair with adenosine instead of cytosine during replication, promoting GC/TA transversion mutations. Thus, 8-OHdG is used as a biomarker of oxidative stress, aging, and carcinogenesis (12).

Early diagnosis of oral cancer by biomarkers related to carcinogenesis is a constant objective, and the development of technologies such as porTable enzymatic detection (e.g., OFNASET) has encouraged researchers to preferentially use saliva over other fluids as a sample. For example, 8-OHdG has only been evaluated in fluids such as saliva and plasma with the objective of investigating it as a biomarker for potentially malignant transformation from premalignant lesions (13), where cases of squamous cell carcinoma of the head and neck (14-17) have been used only as a comparative group. To date, only Hamidavi *et al*. have evaluated oral cancer clinical characteristics in the presence of 8-OHdG, but they performed DNA tissue extractions. However, to determine whether 8-OHdG can be used as a predictive marker of malignant characteristics in cells, we considered the immunoexpression assay to be the best option since it has been widely used for diagnosis, prognosis, and response to cancer therapy (18,19). In addition, tissue 8-OHdG immunoexpression and its possible association with predictive characteristics are unknown in oral cancer.

This work aimed to evaluate the intensity of 8-OHdG immunoreactivity and identify its positivity in cell structures (cytoplasm/nucleus) in OSCC tissues. In addition to grouping intensity/positivity, 8-OHdG immunoreactivity was associated with the presence of risk habits (smoking and drinking) and with prognostic clinical data, such as TNM stage, clinical stage, therapy response and death.

## Material and Methods

In a cross-sectional study of patients diagnosed with OSCC who attended the Jalisco Institute of Cancerology in the state of Jalisco, Mexico (Coronel Calderón 715, El Retiro, 44280 Guadalajara, Jal.) and were evaluated in the Surgical Head and Neck Oncology Department clinically and by computerized tomography, we collected paraffin-embedded tissue samples previously used for diagnostic purposes from patients who attended from 2013 to 2018. Next, patients were grouped according to the WHO histopathological grading system (20), described in the histopathological report. Owing to the retrospective nature of the study, it was exempted from ethical clearance by the institutional ethics committee; however, written informed consent was obtained from all participants.

Information about risk habits, recurrence, persistence, type of therapy and death were taken from patients’ medical history. The patient must have had at least 3 years of follow-up. In an effort to address potential sources of bias, disease stage had to be determined according to the TNM system based on AJCC (21).

Disease-free survival (DFS) was defined as the time from surgery to histologically confirmed recurrence or death from any cause in patients without recurrence. Overall survival (OS) was defined as the time from surgery to the time of death from any cause. Survival curves were plotted using the Kaplan-Meier method and compared using long-rank test.

- 8-hydroxy-2´-deoxyguanosine antibody immunoassay

For the immunohistochemical analyses, 2-μm sections were treated with a heat retrieval solution (Reveal Decloaker, RTU; Biocare Medical, Pacheco, CA, USA) to expose the antigenic epitopes. The endogenous peroxidases were blocked with 0.9% hydrogen peroxide for 5 min. The tissue sections were incubated with a primary antibody against 8-OHdG at room temperature (1:100 dilution, Santa Cruz Biotechnology, catalog number: SC-66036, Santa Cruz, CA, USA) for 60 min and then incubated with a biotinylated anti-mouse/anti-rabbit antibody and a streptavidin-horseradish peroxidase complex at room temperature for 40 min each (Mouse/Rabbit Immuno-detector Biotin Link & HRP Label; Bio SB, Santa Barbara, CA, USA). The reaction products were visualized using the 3,3’-diaminobenzidine-H2O2 substrate (Biocare Medical), and the sections were counterstained with Mayer’s hematoxylin. Control renal, lymphoid and oral mucosa tissue samples were used, and a healthy oral mucosa sample was used as a control for all subsequent reactions.

- Intensity of immunoreactivity evaluation and positivity

Next, immunoexpression was grouped by an oral pathologist according to the following semiquantitative scores:

Negative: negative immunohistochemical staining or positive immunohistochemical staining of <5% of the cells

Weak-moderate: staining of 5 to 50% of the cells

Strong: staining of >50% of the cells.

Positive staining in the cytoplasm/nucleus and the mesenchymal/stromal components was recorded as “present” or “absent”.

- Statistical analyses

The data were transferred to the SPSS Statistics for Windows statistical package database, version 20. Categorical variables are presented as numbers or percentages and were compared using Fisher’s exact test because all expected counts were 5, and 95% confidence intervals (95% CIs) were used for the comparison between immunoexpression intensity and cell structure groups, adopting a statistical significance threshold of *p value* 0.05. The phi coefficient, also called the mean square contingency coefficient, was used as a measure of association. Survival curves was plotted using the Kaplan-Meier method and compared using long-rank test.

## Results

We collected 30 samples from patients seen in the oncology head and neck department and stained them with 8-OHdG antibody. In all patients from this cohort, surgery was the principal treatment, and depending on extension to vital structures and tumor size, chemotherapy was administered as an adjuvant or concomitant treatment with radiotherapy. The chemotherapeutics used were cisplatin and 5-fluorouracil; radiotherapy was administered hypo- or hyperfractionated.

A total of 17 (56.7%) tissue samples were taken from men, and 13 (43.3%) were taken from women, with a mean age of 63.0±12.3 years old. We recorded the following information from the clinical history. A total of 16 (53.3%) smoked, and 13 (46.7%) drank. OSCC localization was more frequent in the tongue, found in 9 (30.0%) cases. Regarding TNM and clinical stage, we observed that most patients had tumors larger than 4 cm (25, 83.3%), slightly more than half (17, 50%) presented with regional metastasis, and distant metastasis was found in only one patient in this group. The average follow-up was 5.4 1.3 years, with a minimum of 3.3 years and a maximum of 7.2 years during this period, revealing that the majority did not report DFS (23, 76.7%). Four patients died during this period (Table 1).

Regarding tumor differentiation, most cases (23, 76.6%) were considered completely well differentiated, followed by moderate (6, 20.0%), and only one case (3.3%) was poorly differentiated.

- 8-hydroxyguanosine immunoexpression

**Figure 1:**
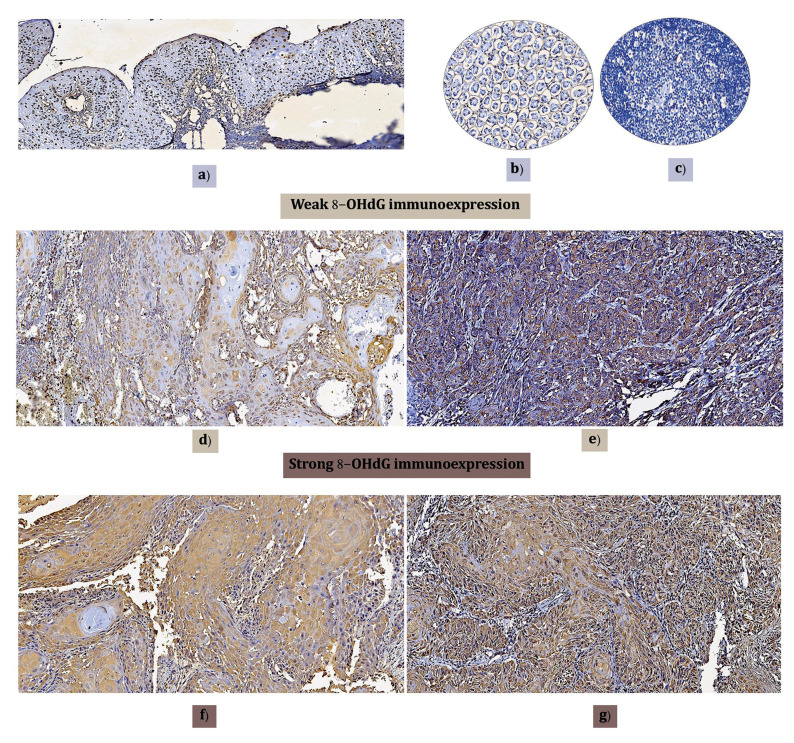
Immunoexpression of 8-OHdG intensity. a) Positive control. Healthy oral mucosa with nuclear positivity immunoexpression. b and c) Negative control. Renal and lymphoid tissue with negative expression. d) Well-differentiated oral carcinoma with weak 8-OHdG immunoexpression, preferentially negative in the squamous differentiation transition area. e) Poorly differentiated oral carcinoma with weak immunoexpression. f) Well-differentiated oral carcinoma with strong 8-OHdG immunoexpression but negative or weak immunoexpression in squamous differentiation transition areas. g) Moderately differentiated oral carcinoma with strong 8-OHdG immunoexpression (Magnifications 10x).

**Figure 2:**
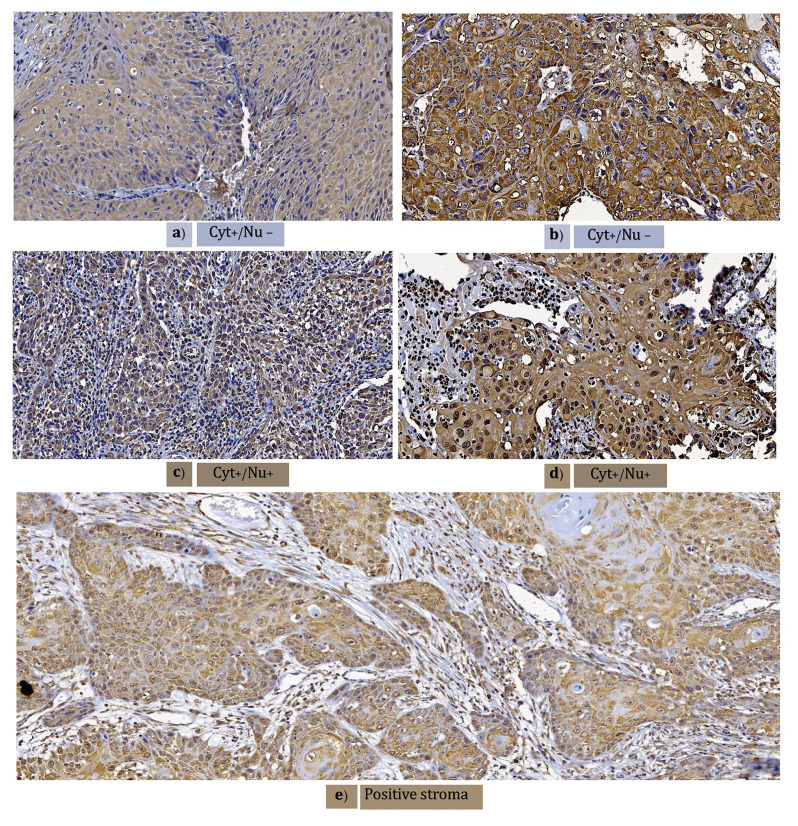
Cellular structure immunoexpression and stroma. Weak (a) or strong (b) cytoplasmic positivity for 8-OHdG immunoexpression with nuclear negativity. Weak (c) or strong (d) cytoplasm and nuclear 8-OHdG immunoexpression positivity. e) Positive 8-OHdG immunoexpression stroma (Magnifications 10x).

Oral mucosa control tissues showed nuclear positivity (Fig. 1), but renal and lymphoid control tissues were negative (Fig. 1). The tumoral parenchyma was included less frequently, with weak intensity (6, 20.0%) in the tissue samples. Most of the cases expressed 8-OHdG with a strong intensity (24, 80%). Regardless of weak (Fig. 1) or strong (Fig. 1) 8-OHdG expression, negative cells with squamous transitions or keratin pearls were always observed. No tissue was classified as completely negative. In addition, almost all samples preferentially stained only in the cytoplasm (21, 70.0%) (Fig. 2), but nuclear positivity was found in 9 (30.0%) OSCC tissues (Fig. 2), independent of the intensity (Fig. 2). In 26 (86.6%) of the cases, the stroma was positive with dying nuclei and cytoplasm of fibroblasts (Fig. 2). Further analysis was performed to compare risk habits, disease clinical characteristics, therapy response and death data between the groups by intensity, and we also clustered the samples based on nuclear and cytoplasm positivity or cytoplasm positivity only (Table 2).

**Table 1:**
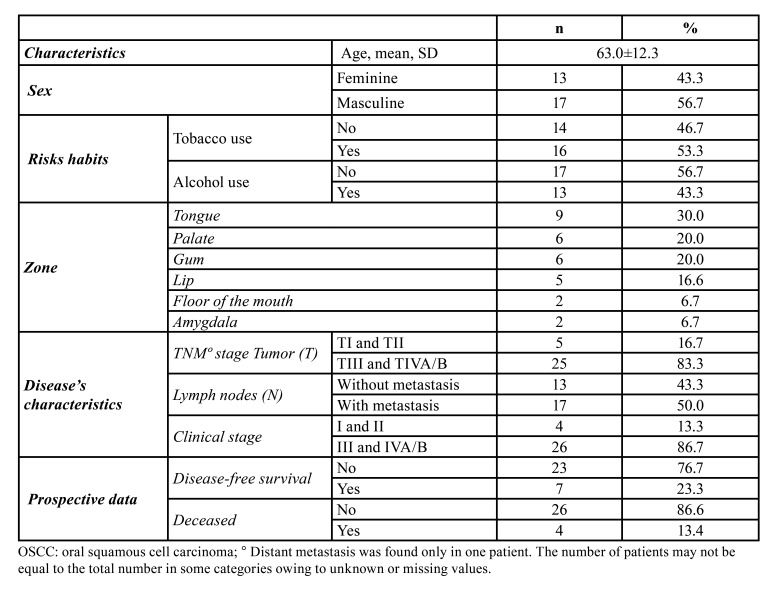
Descriptive Characteristics of patients with OSCC (*n*=30).

**Table 2:**
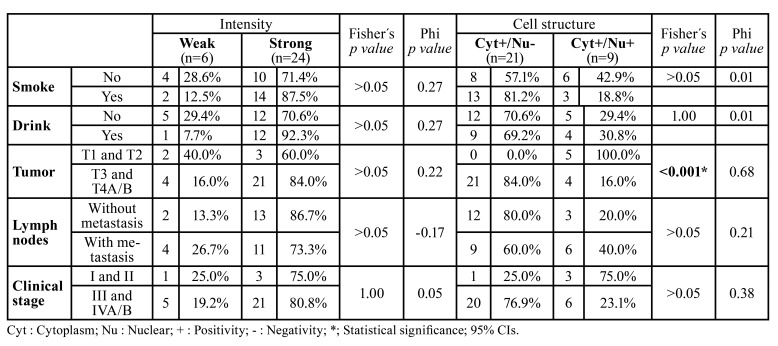
Distribution and significance according 8-hydroxyguanosine immunoexpression (*n*=30).

- Risk habits and 8-OHdG expression

No significant association with risk habits was found, although we more frequently observed strong intensity staining in smokers (14, 87.5%) in contrast to nonsmokers, who mostly displayed weak 8-OHdG staining (4, 28.6%); this difference was not statistically significant (*p value* >0.05). However, no statistical significance was found when this habit was evaluated according to cytoplasm vs. nucleus staining because only cytoplasm staining was markedly frequent in smokers (13, 81.2%) (*p value* >0.05). Drinkers showed an equal distribution of strong intensity compared with nondrinkers, with no significant differences (*p value* >0.05). However, as shown in Table 2, nondrinkers more frequently had negative nuclear staining (12, 70.6%) than drinkers, without statistical significance (*p value*= 1.00).

- Association between TNM stage and clinical stage with 8-OHdG expression

Tumor size: Tumors larger than 4 cm (T3 and T4A/B) strongly expressed 8-OHdG (21, 84.0%), without statistical significance (*p value* > 0.05) compared with smaller tumors. Surprisingly, we observed that all cytoplasmic 8-OHdG expression occurred in tissues >4 cm (21, 84.0%), with a statistically significant difference (*p value* <0.001) (Table 2).

Lymph node invasion: There was no significant difference in the frequency distribution according to the intensity of 8-OHdG expression between lymph nodes with and without invasion (*p value* >0.05). Additionally, there was no predilection based on nuclear positivity (*p value* >0.05) (Table 2).

Clinical stage: As expected, because large tumors and lymphatic node invasion presented with a higher frequency of strong 8-OHdG expression, samples with a clinically advanced stage (III and IVA/B) had the largest frequency concentration (80.8%), without statistical significance (*p value*= 1.00). However, these clinically advanced cases frequently had cytoplasm staining with negative nuclear 8-OHdG expression (76.9%), without statistical significance (*p value* >0.05).

- Disease-free survival and overall survival

**Figure 3:**
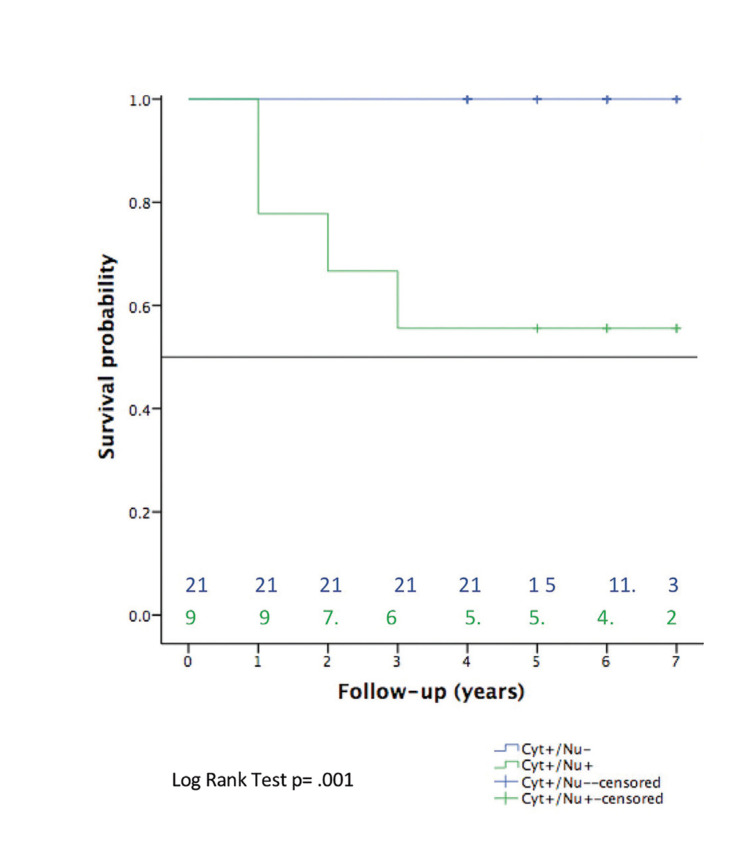
Kaplan‒Meier estimator and number at risk for overall survival according to cell structure immunoexpression. Green arrow indicates near of 50% survival. Blue arrow remains at 100%.

DFS and OS were estimated using Kaplan‒Meier estimators and the log-rank test for comparisons between groups. Kaplan‒Meier curves and log-rank tests did not reveal significant differences in either intensity (*p value* >0.05) or cell structure (*p value* >0.05) for DFS. Finally, to determine the OS influence of 8-OHdG immunoexpression, we again applied Kaplan‒Meier estimators and the log-rank test. The results were not significantly different according to OS and intensity (*p value*= 0.05), but in patients with double-positive (cytoplasm and nucleus) immunoexpression a significant difference (*p value* = 0.001) was founded, falling close to 50% the as shown in Fig. 3. Some highlights are interesting; all deceased patients belonged to this group, and shared characteristics, such as female sex and no risk habits.

## Discussion

Oral cancer is a global health problem and is one of the most important causes of mortality. It is a very aggressive and severe pathology that has had a greater impact on the population in recent years. In 2020, GLOBOCAN reported an increase in the number of cases of carcinoma in the lip and oral cavity in both sexes in people of legal age, estimating an incidence number of cases in 2040 of 545,396. Due to its effect on human health and the relentless search to find and develop a cure, cancer has been the focus of a great research effort for many years. Such investigations have achieved remarkable advances in understanding the cellular and molecular bases but have had little impact on preventing tumor development or increasing the probability of survival of most cancerous tumors (3,2).

Tumor cells generate higher levels of ROS than normal cells due to the high metabolic rate and loss of normal mitochondrial function, increasing susceptibility to free radicals and thus oxidative stress and suggesting that ROS are a biomarker of tissue malignancy (22).

Multiple studies have sought and answered questions about oxidative stress related to oral premalignant lesions (OPLs) and OSCC. For example, Shengore *et al*. (2018) demonstrated that in peripheral blood, patients with OPL (leukoplakias) maintained higher levels of 8-OHdG than a control group. Babiuch *et al*. (2019) and Nandankumar *et al*. (2020) evaluated this free radical in saliva from patients with OSCC. Babiuch found that there were no significant differences in the levels of 8-OHdG compared to a control group. In contrast, Nandankumar found high levels of 8-OHdG, demonstrating significant differences in the concentration of salivary 8-OHdG among healthy controls, oral submucous fibrosis patients and OSCC patients (13,16,17).

These controversial results might have occurred because body fluid samples and tissue homogenates can indiscriminately mix several cell types that are susceptible to generating this radical (such as leukocytes and fibroblasts).

To the best of our knowledge, this study is the first to evaluate 8-OHdG immune expression in OSCC tissues, where we can demonstrate DNA oxidative damage at the parenchyma level. According to our literature search, only three studies on other types of cancer have performed 8-OHdG immunoassays. In accordance with these findings, immunoreactivity was found in all carcinoma samples: 110 hepatocellular carcinomas (23), 8 papillary thyroid samples (24), and 10 prostatic adenocarcinoma samples (25).

We expected that the difference would be reflected according to the intensity of immunoreactivity, and significance would be related to these parameters, as observed in previously cited studies. Although this study showed a high intensity of 8-OHdG immunoreactivity in OSCC tumors, the significance of some criteria (tumors and deaths) was based on cytoplasmic/nuclear immunoexpression.

According to the results of this study, the presence of risk factors does not affect the expression of this free radical, contrary to the strong association established between tobacco and alcohol consumption and the induction of ROS. This lack of association could be explained by the endogenous oxidative damage (EOD) resulting in 8-OHdG being caused independently of those risk factors (6), such as through alterations related to glucose (26), the presence of inflammation, and the maintenance of tumorigenesis, which in turn promotes a high metabolic energy demand (27,28). Consequently, homeostatic imbalance between antioxidants and free radicals remains in the tumor microenvironment.

In thyroid papillary carcinomas (24), the production of ROS has been demonstrated to be an endogenous source from the cancer cells themselves involving the progression of cancerous conditions to more severe stages. According to this finding, EOD is a phenomenon shared between papillary carcinomas and oral cancer.

In concordance with Ohtake *et al*. (2018), the significantly preferential cytoplasmic immunoexpression of OSCC tissues was part of our results. But we cannot consider this is a robust finding, in first place the lack of inclusion of OPLs, one limitation of our study, prevents make more hypotheses related to tumor growth.

The differences found regarding the location of the immunomarker (cytoplasm and nucleus), drives perspectives, about exploring the mechanism by which malignant cells shield the nucleus to protect nuclear DNA from 8-OHdG damage. In this sense, it can be hypothesized that more oxidative damage occurs when the cytoplasm and nucleus express 8-OHdG, which would agree with the significant associations of 8-OHdG cytoplasm/nucleus expression with all OSCC deaths found in this study. Supporting this, Li *et al*. (2012) followed patients with hepatocellular carcinoma for 3 years and concluded that lower survival rates were associated with high 8-OHdG immunoexpression. It would be necessary to corroborate the oxidative progress from the cytoplasm to the nucleus for this outcome, so more studies are needed to reinforce this approach. It is interesting to consider how tumor cells can develop mechanisms to continue functioning despite being subjected to oxidative damage, unlike normal cells that can do so with low levels of ROS.

These hypotheses are the origin of the pro-oxidant or antioxidant therapy controversy, because antioxidant therapy can protect against the toxic effects of ROS and accelerate cancer progression, and the elevation of ROS redox could also be useful for cancer establishment (12). Therefore, more knowledge about malignant cell behavior during oxidative stress is necessary.

- Limitations

Among the limitations of our study was the small sample size; we only examined a piece of the total tumor, and we cannot know whether oxidative stress characteristics were present in the complete tumor. Additionally, for normal oral tissue nuclear positivity, it was not possible to determine the reason for this result because the donors’ medical histories were unknown and because data such as old age, presence of risk habits or comorbidities related to oxidative damage were not available.

## Conclusions

DNA oxidative damage assessment by 8-hydroxyguanosine immunoexpression is possible in oral cancer tissues but is not related to risky habits. However, this study demonstrated the oxidative state with a biomarker that allows the visualization of important cell structures, such as the nucleus. Additionally, in OSCC, we observed preferential cytoplasmic staining alone or in conjunction with nuclear staining. Associations with predictive disease characteristics, such as tumor size and overall survival, were observed, suggesting that high oxidative damage is present when confined to the cytoplasm and nucleus.

## References

[1] Mattiuzzi C, Lippi G (2019). Current Cancer Epidemiology. J Epidemiol Glob Health.

[2] Pratima K, Priyanka D, Anshuman D (2022). Oral potentially malignant disorders: etiology, pathogenesis, and transformation into oral cancer. Front Pharmacol.

[3] Ferlay J, Soerjomataran I, Dikshit R, Eser S, Mathers C, Rebelo M (2015). Cancer incidence and mortality worldwide: sources, methods and major patterns in GLOBOCAN 2012. Int J Cancer.

[4] d'Alessandro AF, Pinto RF, Lin CS, Vamondes-Kulcsar MA, Cernea CR, García-Brandão L (2015). Oral cavity squamous cell carcinoma: factors related to occult lymph node metastasis. Braz J Otorhinolaryngol.

[5] Hanahan D, Weinberg RA (2011). Hallmarks of cancer: the next generation. Cell.

[6] Valavanidis A, Vlachogianni T, Constantinos F (2009). 8-hydroxy-2′ -deoxyguanosine (8-OHdG): A critical biomarker of oxidative stress and carcinogenesis. J Environ Sci Health C Environ Carcinog Ecotoxicol Rev.

[7] Lunec J, Holloway KA, Cooke MS, Faux S, Griffiths HR, Evans MD (2002). Urinary 8- oxo-2 -deoxyguanosine: redox regulation of DNA repair in vivo?. Free Radic Biol Med.

[8] Valko M, Izakovic M, Mazur M, Rhodes CJ, Telser J (2004). Role of oxygen radicals in DNA damage and cancer incidence. Mol Cell Biochem.

[9] Kasai H (2002). Chemistry-based studies on oxidative DNA damage: Formation, repair, and mutagenesis. Free Radical Biology and Medicine.

[10] Cheng KC, Cahill DS, Kasai H, Nishimura S, Loeb LA (1992). 8-Hydroxyguanine, an abundant form of oxidative DNA damage, causes G----T and A----C substitutions. J Biol Chem.

[11] Halliwell B, Ahikary A, Dingfelder M, Dizdaroglu M (2021). Hydroxyl radical is a significant player in oxidative DNA damage: In vivo. Chem Soc Rev.

[12] Martin KR, Barrett JC (2002). Reactive oxygen species as double-edged swords in cellular processes: low-dose cell signaling versus high-dose toxicity. Hum Exp Toxicol.

[13] Shenghore T, Li FY, Sung FG, Tsai MH, Hua CH, Liu CS (2018). Biomarkers of oxidative stress associated with the risk of potentially malignant oral disorders. Anticancer Res.

[14] Bahar G, Felnmesser R, Shpitzer T, Popovtzer A, Nagler RM (2007). Salivary analysis in oral cancer patients: DNA and protein oxidation, reactive nitrogen species, and antioxidant profile. Cancer.

[15] Kumar A, Pant MC, Singh HS, Khandelwai S (2012). Determinants of oxidative stress and DNA damage (8 OhdG) in squamous cell carcinoma of head and neck. Indian J Cancer.

[16] Babiuch K, Bednarczyk A, Gawlik K, Pawlica-Gosiewska D, Kęsek B, Darczuk D (2019). Evaluation of enzymatic and non-enzymatic antioxidant status and biomarkers of oxidative stress in saliva of patients with oral squamous cell carcinoma and oral leukoplakia: a pilot study. Acta Odontol Scand.

[17] Nandakumar A, Priyadharsini N, Amritha J, Rajkumar K, Mahesh KM (2020). Estimation of Salivary 8-Hidroxydeoxyguanosine (8-OhdG) as a potential biomarker in assessing progression towards malignancy: A case-Control Study. Asian Pac J Prev.

[18] Broggi G, Filetti V, Ieni A, Rapisarda V, Ledda C, Vitale E (2020). MacroH2A1 immunoexpression in breast cancer. Front. Oncol.

[19] de Freitas Filho SAJ, Coutinho-Camillo CM, Oliveira KK, Bettim BB, Pinto C AL, Kowalski LP (2021). Prognostic Implications of ALDH1 and Notch1 in different subtypes of oral cancer. J Oncol.

[20] Lindenblatt Rde C, Martinez GL, Silva LE, Faria PS, Camisasca DR, Lourenço Sde Q (2012). Oral squamous cell carcinoma grading systems--analysis of the best survival predictor. J Oral Pathol Med.

[21] Lins LS, Bezerra NV, Freire AR, Almeida LD, Lucena EH, Cavalcanti YW (2019). Socio-demographic characteristics are related to the advanced clinical stage of oral cancer. Med Oral Patol Pral Cir Bucal.

[22] NavaneethaKrishnan S, Rosales J, Lee K (2018). Targeting Cdk5 for killing of breast cancer cells via perturbation of redox homeostasis. Oncoscience.

[23] Li S, Wang X, Wu Y, Zhang H, Zhang L, Wang C (2012). 8-Hidroxy-2'deoxyguanosine expression predicts hepatocellular carcinoma outcome. Oncology Letters.

[24] Mseddi M, Mansour RB, Gouiia N, Mnif F, Bousselaa R, Abid M (2017). Comparative study of nuclear 8-hidroxyguanosine expression in autoinmmune Thyroid Diseases and Papillary Thyroid Carcinoma and its relationship with p53, Bcl-2 and ki-67 cancer related proteins. Advances in Medical Sciences.

[25] Ohtake S, Kawara T, Ishiguro Y, Takeshima T, Kuroda S, Izumi K (2018). Oxidative stress marker 8-hydroxiguanosine is more highly expressed in prostate cancer than in benign prostatic hyperplasia. Molecular and Clinical Oncology.

[26] Chhipa AS, Borse SP, Baksi R, Lalotra S, Nivsarkar M (2019). Targeting receptors of advanced glycation end products (RAGE): preventing diabetes induced cancer and diabetic complications. Pathol Res Pract.

[27] Peters A (2006). The energy request of inflammation. Endocrinology.

[28] Pavlova NN, Thompson CB (2016). The emerging hallmarks of cancer metabolism. Cell Metab.

